# Plasmapheresis for Fulminant Wilson's Disease Improves Mental Status and Coagulopathy

**DOI:** 10.1155/2023/3985823

**Published:** 2023-06-19

**Authors:** Quarshie Glover, William Nicholas Rose

**Affiliations:** ^1^Department of Medicine, University of Wisconsin Hospital, 600 Highland Ave, Madison, WI 53792, USA; ^2^Department of Pathology, University of Wisconsin Hospital, 600 Highland Ave, Madison, WI 53792, USA

## Abstract

Wilson's disease is a rare genetic condition that affects copper metabolism, resulting in tissue copper accumulation and resultant organ damage. We report a case of a young woman who presents with Wilson's disease complicated by hemolysis, impaired hepatic function, coagulopathy, and acute kidney injury. She was treated with plasmapheresis as a bridge to a liver transplant. Her mental state, renal function, and bilirubin level improved after starting plasmapheresis. She successfully underwent a liver transplant and remained stable post-liver transplant. We share our experience on the use of plasmapheresis in treating Wilson's disease.

## 1. Introduction

Wilson's disease causes copper accumulation in tissues such as the liver, brain, cornea, and kidneys. This results in organ impairment and subsequent damage. Copper is also toxic to red blood cells, resulting in hemolysis. Plasmapheresis is a treatment option for managing Wilson's disease in the setting of complications as a bridge to a liver transplant. Plasmapheresis involves the removal of copper and other toxic metabolites like ammonia from the bloodstream, thus ameliorating the harmful effects of excessive copper accumulation in the body [1]. We share our experience with using plasmapheresis to manage Wilson's disease within a specific clinical context.

## 2. Case Presentation

The patient is a 17-year-old female who presents with a 5-day history of abdominal pain, nausea, vomiting, diarrhea, fatigue, headache, and jaundice 24 hrs prior to admission. She denies alcohol intake and the use of recreational drugs. Examination showed temp: 37°C (98.6°F), pulse: 96, resp: 16, and SpO_2_: 98% on room air. She was frankly jaundiced and had scleral icterus; she appeared drowsy and mildly distressed. She had right upper quadrant tenderness and generalized abdominal tenderness. Blood tests showed mild AST elevation 88 U/L (2 times the upper limit of normal), normal ALT 13 U/L, low alkaline phosphatase (26 U/L (N: >50)), mildly elevated GGT (56 U/L (N: <40)), and elevated total bilirubin (32.6 mg/dl (N: <1.4)) with a conjugated form of 24.3 mg/dl (N: <0.3), elevated INR 2.4 (N: 0.9–1.1), albumin 2.3 g/dl (N: 3.3–4.7), and activated PTT 53.9s (N: 26–35). A complete blood count demonstrated low hemoglobin 7.7 g/dl (N: 12–15), low platelet 139 k/ul (N:160–370), and elevated white cell count 28.6 k/ul, (N: 4–10.5). Anemia was due to hemolysis as demonstrated by low haptoglobin <8 mg/dl (N: 30–200), high LDH 420 U/L (N: 90–245), and an elevated reticulocyte count of 11.4% (0.5–2.2) in the absence of bleeding. The direct Coombs test was negative, showing that hemolysis was likely not antibody-mediated. Hemolysis was due to elevated serum copper [2]. The patient also had acute kidney injury with creatinine 1.7 mg/dl (0.55–1.05). Acute kidney injury was due to the harmful effects of copper on renal function [3, 4]. The patient also had volume loss from diarrhea and vomiting, which may have contributed to renal impairment. Other laboratory investigations showed serum copper 175 *μ*g/dl (N: 57–129), urine copper >250 *μ*g/dl (N: 0.2–8), and ceruloplasmin 9 mg/dl (20–55). Negative infectious workup for Hep A, Hep B, Hep C, adenovirus, HIV, EBV, and CMV serum PCRs. Some autoimmune etiologies of liver injury were ruled out with negative ANA, smooth muscle antibodies, antimitochondrial antibodies (PBC), and microsomal antibodies. Deep Doppler abdominal ultrasound showed an enlarged spleen, gall bladder sludge without evidence of cholecystitis, no biliary tract disease, and no hepatic vascular lesions. Her presentation was thought to be likely due to Wilson's disease due to elevated serum and urine copper levels and low ceruloplasmin. A slit lamp examination did not show Kayser–Fleischer rings. The patient was treated with IV fluids, antiemetics, morphine for pain control, vitamin K, Epogen, and furosemide as needed. On the second day of admission, she underwent plasmapheresis.

**Figure 1:**
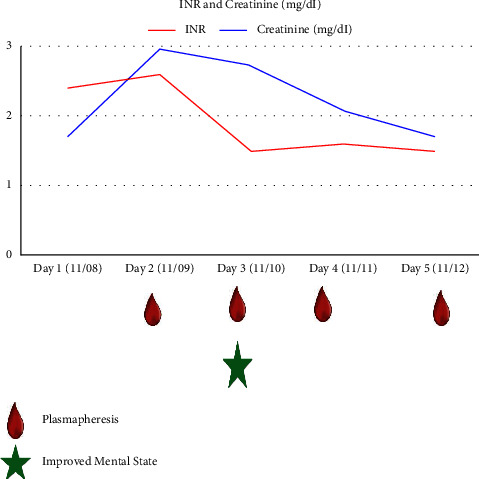
Improved INR, creatinine, and mental state after starting plasmapheresis.

Each plasmapheresis was performed via centrifugation and exchanged for one plasma volume using plasma as the replacement fluid. After plasmapheresis, examination showed improvement in her mentation (Figure 1). The patient was reported to be awake, alert, and interactive. She also had a decrease in INR (Table 1). Liver enzymes remained stable. A liver biopsy on the third day of admission showed a tissue copper level of 447 mcg/g dry weight [N: 10–35].

Histology of liver biopsy obtained via the transjugular route showed cholestasis, moderate microvesicular steatosis, focal ballooning of hepatocytes, rare glycogenated nuclei, and bile ductular proliferation. Trichrome and reticulin stains highlight pericellular fibrosis and parenchymal necrosis/collapse. She continued on daily plasmapheresis and underwent a liver transplant on day 6 of admission. She was subsequently started on immunosuppressive therapy posttransplant and discharged home in stable condition. Due to the short interval between the first plasmapheresis and transplant, no postplasmapheresis copper levels were obtained.

## 3. Discussion

This case illustrates the use of plasmapheresis for the treatment of Wilson's disease and its associated complications prior to a liver transplant. Clinical manifestations of Wilson's disease result from copper accumulation in various tissues and organs [5]. Patients may present with lethargy, anorexia, jaundice, abdominal pain, intellectual deterioration, recurrent epistaxis, hepatomegaly, and splenomegaly [6]. Other clinical manifestations include neurological deficits, liver injury, kidney injury, and hemolysis. Liver injury may present in various forms, ranging from asymptomatic liver function test abnormalities to hepatitis, acute liver failure, and cirrhosis [7]. Our patient presented with mildly elevated liver enzymes, with AST being twice the upper limit of normal. The ALT level was within normal limits. Serum alkaline phosphatase was low, which is a common finding [8]. There was a decrease in albumin and an elevated INR, demonstrating impaired synthetic function of the liver. She also had acute kidney injury and intravascular hemolysis, likely due to copper accumulation and the effect of copper on red blood cells. Wilson's disease can result in severe disability and death; thus, prompt and adequate treatment is needed to prevent clinical deterioration and mortality [8]. Chelating therapy is an effective treatment option for Wilson's disease [9]. However, our patient did not undergo chelating therapy, as it was thought that in acute decompensation, chelation therapy is not indicated and would worsen her kidney function. In her case, the prognosis was also deemed to be poor without liver transplantation. This resulted in an expedited liver transplant on the 6th day of admission. In the interim, plasmapheresis was done. Her clinical condition improved after starting plasmapheresis, as evidenced by improved mental state (interactive, awake, and alert), renal function, and INR. Due to the fact that serum, tissue, and urine copper levels were obtained once, improvement in these levels could not be monitored after plasmapheresis. Studies have shown that plasmapheresis may improve transplant-free survival for longer periods [10], with some patients recovering without a liver transplant [11, 12]. Notably, plasmapheresis with chelating therapy has been beneficial in cases of Wilson's disease that did not require a liver transplant. Adding to the body of knowledge and literature on the use of plasmapheresis for the management of Wilson's disease will shed more light on this very vital treatment modality that can be improved. The characteristics of the patients that were able to undergo plasmapheresis without needing a liver transplant is also an important aspect of management that needs further research. Predictive scoring modalities may be may be helpful in triaging patients that will benefit most from one treatment modality.

## 4. Conclusion

We share our experience of a patient who presents with Wilson's disease associated with lethargy, hemolysis, impaired hepatic function, coagulopathy, and acute kidney injury. She received daily plasmapheresis starting on the second day of admission for five days till liver transplant. There was an improvement in the patient's sensorium, INR, and serum creatinine. Plasmapheresis remains an important treatment option for preventing rapid clinical deterioration and worsening complications associated with Wilson's disease, which patients often present with. It continues to serve as a bridge to liver transplant and may be used for longer periods in certain clinical contexts in association with other therapeutic interventions like chelating agents, as liver transplants may not be readily available or patients may not meet the criteria for an immediate liver transplant.

## Figures and Tables

**Table 1:** Table showing improvement in INR and serum creatinine after starting plasmapheresis.

	INR	Creatinine (mg/dl)
Day 1 (11/08)	2.4	1.7
Day 2 (11/09) ∗	2.6	2.96
Day 3 (11/10)	1.5	2.72
Day 4 (11/11)	1.6	2.09
Day 5 (11/12)	1.5	1.68

## Data Availability

The data used to support the findings of this study were obtained from patients charts (confidentiality and HIPPA observed).

## Abbreviations

ANA: Antinuclear antibodies
AST: Aspartate aminotransferase
ALT: Alanine aminotransferase
CMV: Cytomegalovirus
EBV: Ebstein Bar virus
GGT: Gamma-glutamyl transferase
Hep A: Hepatitis A
Hep B: Hepatitis B
Hep C: Hepatitis C
HIV: Human immunodeficiency virus
INR: International normalized ratio
LDH: Lactate dehydrogenase
PBC: Primary biliary cirrhosis
PCR: Polymerase chain reaction
PTT: Partial thromboplastin time.
